# Proportion of cancer in a Middle eastern country attributable to established risk factors

**DOI:** 10.1186/s12885-017-3304-7

**Published:** 2017-05-18

**Authors:** Maya A. Charafeddine, Sara H. Olson, Deborah Mukherji, Sally N. Temraz, Ghassan K. Abou-Alfa, Ali I. Shamseddine

**Affiliations:** 1 Department of Hematology-Oncology, American University of Beirut Medical Center, P.O Box 11–0236, Riad El Solh, Beirut, 1107 2020 Lebanon; 2 Memorial Sloan-Kettering Cancer Center, Memorial Sloan Kettering Cancer Center, 300 East 66th Street, New York, NY 10065 USA

## Abstract

**Background:**

Providing an estimate of the percentage of cancer in Lebanon by 2018 that is due to the exposure to risk factors in 2008. Factors include: smoking, body mass index (BMI), physical inactivity, dietary factors, alcohol consumption, infections, and air pollution in adults.

**Method:**

Population Attributable Fraction (PAF) was calculated using the proportion of the population exposed and relative risks for each risk factor from meta-analyses. The PAF estimates the proportion of cases in which exposure may have played a causal role.

**Results:**

Smoking caused most cancer cases, and it will further add a total of 1800 new cases by 2018. Among many other cancers, lung cancer had the largest proportion attributable of around 75%. BMI is expected to increase colorectal, liver and gastric cardia carcinoma specifically in males. High physical activity has a an average of 15% protection rate on cancer on colorectal cancer. Minimal adherence to Mediterranean diet will affect gastric cancer incidence by 7%. Cases of oropharyngeal and esophageal cancer will be the result of alcohol consumption mainly in males. H.Pylori infection is expected to result in half of the gastric cases by 2018. The high exposure to air pollution is expected to contribute by 13% to lung cancer cases in 2018.

**Conclusion:**

The highest benefits can be achieved by controlling tobacco smoking. Interrelated and small changes in weight, physical activity and healthy diet with limited alcohol consumption can protect against several GI cancers in the long run. These results can be used to determine public health interventions that target important risk factors in the general population.

## Background

The possibility of developing or dying from cancer is often expressed in population specific incidence and death rates. Alternatively, information on exposure to potential carcinogens over prolonged periods of time can give a glance on the possibility of developing cancer in one’s lifetime [1]. The WHO report estimates that around a third of cancers are caused by several leading environmental and behavioral risk factors, specifically tobacco being the biggest culprit among all causative factors [2].

Changes in exposure to risk factors have played a major role in decreasing cancer mortality rates in lung cancer in developed countries, with a decreasing trend following the decreasing smoking pattern [3]**.** Other pronounced changes in cancer incidence in developed countries were the result of early detection and screening programs such as in colon and breast cancer [4]. In developing countries, however, the previously mentioned cancers are still on the rise, as the adoption of westernized lifestyles is relatively recent compared to developed countries [5]. Migrants’ studies revealed that after a few generations, the incidence rate of migrants’ population becomes comparable to the host country’s population, suggesting that environmental risk factors play a significant role in cancer etiology [5, 6].

Cancer incidence in Lebanon is among the highest in the region and is expected to remain as such over the coming decade, according to a recent national epidemiological study and the ministry of health records [7–9]. Around 9000 cases were reported in 2008 and this number has been increasing by 4–5% annually. Cancer incidence rates are expected to reach 296 and 340 per 100,000 for males and females respectively by 2018 in Lebanon [7], so far the highest recorded cancer incidence among Arab countries. In addition, the World Health Organization estimated 2250 cancer-related deaths in 2012 in Lebanon [10]. While cancer etiology is multifactorial, a set of known risk factors were hypothesized as contributing to the dynamics of cancer epidemiology. The role of specific risk factors will give insight on cancer causation hence providing a quantification of the present risk factors burden [4].

The purpose of this study is to estimate the percentage of cancer in Lebanon that is due to environmental and lifestyle related exposures. Using a new methodologic approach to identify the impact of risk factors in cancer was never performed in the Middle East region, which limited international and regional comparisons. The results can be used to determine public health interventions that target cancer incidence in the general population.

## Methods

Two main components are needed to estimate the role of risk factors in cancer incidence: prevalence of the risk factor in the studied population, and the relative risk of cancer associated with that risk factor. Cancer incidences that are the result of an exposure are presumably related to an accumulation of such exposure over many years, referred to as lag time. A standard latent period of 10 years is considered acceptable for most risk factors [11], although such risk factors are not a point-in-time exposure, many could be present over many years. In this study, the reported exposed population proportion for most risk factors was for 2008; consequently we will estimate the effect of such risk factors on cancer in 2018. The risk factors in this analysis will be restricted to adults.

The selection of risk factors was based on the global health risks report that identified major risk factors that affect disease and mortality. The report identified nine environmental risk factors and seven infectious risk factors that result in nearly half of cancers deaths worldwide [12]. In this report the main risk factors such as smoking, BMI, physical activity, adherence to a healthy Mediterranean diet, and alcohol were included in addition to two other relevant risk factors to the Mediterranean region, including H.Pylori infection and air pollution. All risk factors included in this manuscript are identified as Group 1 carcinogens as per the International Agency for Research on Cancer (IARC), with sufficient evidence in humans [13] (Table 1).

**Table 1:** Risk factors, exposure levels and studied cancer sites

Risk factor	Exposure level	Sources of Data	
		Prevalence of exposure	Relative Risk
Tobacco smoking	Current smoker, former smoker (cessation mean 8.9 years)	Sibai et al. [14] (WHO)	Lung [42] Bladder [43] Larynx [42] Esophagus [42] Stomach [42] Liver [42] Pancreas [42] Cervix [42]
Body Mass Index (BMI)	Overweight (BMI ≥ 25 kg/m^2^) Obese (BMI ≥ 30 kg/m^2^)	Sibai et al. [14] (WHO)	Esophageal and gastric cardia [44] Colon and Rectal [45] Liver [46] Endometrial [47, 48] Renal cell [49] Pancreatic [50] Ovarian [51] Breast [29]
Physical Activity	High (7 days of 3000 MET-minutes/week OR 3 days of 1500 MET-minutes/week) Moderate (≥3 days vigorous intensity, OR ≥5 days moderate intensity, OR ≥5 days ≥600 MET-minutes/week) Low: less than the moderate activity	Sibai et al. [14] (WHO)	Breast [52] Bladder [53] Gastroesophageal [54] Colorectal [55]
Adherence to Mediterranean Diet	9-point scale adherence to Mediterranean diet score Low: 0–3, moderate 4–5, and high 6–9 points.	Using nationally representative dietary intake data of Lebanese adults aged between 20 and 55 years (unpublished data)	Gastric [56] Colorectal [57] Bladder [58]
Alcohol consumption	Heavy: More than 50 g per day	Sibai et al. [14] (WHO)	Oropharyngeal and Esophageal [59] Liver [59] Colon [59] Breast [59]
Infection	Percentage of H. Pylori infections in the Lebanese population	Naja et al. [17]	Gastric [60]
Air pollution (PM_10_ PM_2.5_)	Levels exceeding WHO standards: PM_10_: 20 μg/ m^3^ PM_2.5:_ 10 μg/ m^3^	Saliba et al. [18] Saliba et al. [19]	Lung [40]

*MET*, Metabolic equivalent of task

## Data sources

### Prevalence of exposures

In order to study the impact of each risk factor on the studied population, we relied on population based reports or studies involving a representative sample of the Lebanese population that identified the proportion of the population at risk. The World Health Organization report on chronic disease risk factor surveillance specific to Lebanon included a nationally representative sample of Lebanese adults aged between 25 and 64 from randomly selected households using Lebanese Governorates strata [14]. The surveillance study used standardized questionnaires and relied on physical measurements when applicable. Data on common behavioral factors including tobacco smoking, BMI, physical activity and alcohol consumption were stratified in this report by gender for 2008–2009 [14]. Data on adherence to the healthy Mediterranean diet was retrieved from a nationally representative dietary intake data of Lebanese adults aged between 20 and 55 years [15].The Mediterranean Diet Scale (MDS) was constructed to measure adherence to the Mediterranean Diet using 9 components. Individuals were assigned a score of 1 if their consumption of vegetables, legumes, fruits, cereals, and fish was above the sex specific median intake and 0 otherwise. The opposite scoring was given for meat and dairy. For alcohol, men consuming 10–50 g /day and women consuming 5–25 g /day were given a score of 1 and 0 otherwise. Study participants were grouped according to their score: scores ranging from 0 to 3 (low); scores ranging from 3 to 6 (moderate), and scores ranging between 6 to 9 (high) [16, 17].

Information on H.Pylori infection among the studied population was retrieved from a nation-wide cross-sectional study involving the adult population in Lebanon in 2010 [18]. Numerous studies on air pollution were conducted between 2004 and 2007 measuring particulate matter (PM_10_ and PM_2.5_) concentrations at different points around Greater Beirut area in Lebanon, in which more than two thirds of the Lebanese population resides [19, 20]. Measurements of air pollution were taken over 1 year for 24 h every 6 days, in a representative heavily populated area with high traffic density in Beirut. The World Health Organization PM_10_ and PM_2.5_ annual averages are 20 μg/ m^3^ and 10 μg/ m^3^ respectively [19, 20].

The proportion of the Lebanese population with all the risk factors were reported for 2008, except for air pollution (2004–2007) and H.Pylori infection (2010). Noting that no major changes such as outbreaks occurred in this period, it is safe to assume that this data can be extrapolated for 2008. Table 2 summarizes the prevalence of each of the mentioned risk factors by gender.

**Table 2:** Prevalence of risk factors in Lebanon in adult males and females aged 25–65 years

Risk Factor	Males	Females
Tobacco smoking, [14]		
former smokers	6.9%	3.3%
current smokers	46.8%	31.6%
Body Mass Index (BMI) [14]		
overweight	30.7%	38.9%
obese	28.7%	26.5%
Physical activity, Low activity [14]	52.4%	40.3%
Low adherence to Mediterranean diet [15]	14.4%	12.3%
Alcohol [14]		
Heavy drinkers	12.0%	0.9%
H. Pylori infection* [18]	52%	52%
Air pollution* (PM_10_ and PM_2.5_concentrations above allowable levels) [19, 20]	85%	85%

*PM* Particulate matter

To complete the analysis, relative risks were retrieved for each risk factor from meta-analyses from epidemiological studies, as shown in Table 1. Meta-analyses are valuable as they represent large populations by pooling the results and identifying patterns from many studies. Criteria for meta-analyses selection included meta-analyses that adopted original articles of case control and cohort studies with an assessment of research quality if available. Assuming that risk related cancers are sufficiently rare, we adopted the use of OR and RR equally. Preference was given for papers including RR stratified by gender and in risk factors’ sub-categories used in this article. Relative risks derived from these meta-analyses are shown in Table 3.

**Table 3:** Derived relative risks (RR) from meta- analyses used in the PAF estimation

Risk Factor	Cancer site	Males	Females	References
Tobacco smoking (compared to non-smokers)	Esophagus	Current smokers: 2.5 Previous smokers: 2.03	Current smokers: 2.5 Previous smokers: 2.03	[42]
	Larynx	Current smokers: 6.98 Previous smokers: 2.31	Current smokers: 6.98 Previous smokers: 2.31	[42]
	Lung	Current smokers: 8.96 Previous smokers: 3.85	Current smokers: 8.96 Previous smokers: 3.85	[43]
	Bladder	Current smokers: 3.3 Previous smokers: 2.0	Current smokers: 3.1 Previous smokers: 1.65	[42]
	Stomach	Current smokers: 1.64 Previous smokers: 1.31	Current smokers: 1.64 Previous smokers: 1.31	[42]
	Liver	Current smokers: 1.56 Previous smokers: 1.49	Current smokers: 1.56 Previous smokers: 1.49	[42]
	Pancreas	Current smokers: 1.71 Previous smokers: 1.18	Current smokers: 1.71 Previous smokers: 1.18	[42]
	Cervix		Current smokers: 1.83 Previous smokers: 1.26	[42]
Body Mass Index (BMI) (compared to BMI ≤ 25 kg/m^2^)	EGCA	Overweight:1.71 Obese: 2.34	Overweight:1.71 Obese: 2.34	[44]
	Colon	Overweight:1.26 Obese: 1.71	Overweight:1.09 Obese: 1.1	[45]
	Rectum	Overweight:1.07 Obese: 1.75	Overweight:1 Obese: 1.12	[45]
	Liver	Overweight:1.3 Obese: 1.8	Overweight:1.04 Obese: 1.63	[46]
	Endometrium		Overweight:1.02 Obese: 1.19	[47]
	Renal cell	Overweight:1.22 Obese: 1.63	Overweight:1.38 Obese: 1.95	[49]
	Pancreas	Overweight:1.1 Obese: 1.5	Overweight:1.1 Obese: 1.5	[50]
	Ovary		Overweight:1.08 Obese: 1.27	[51]
	Breast		Overweight:1.02 Obese: 1.19	[29]
Physical Activity (low activity compared to medium and high activity) Table 1	Breast		Low:1.16	[52]
	Bladder	Low:1.09	Low:1.20	[53]
	Gastric-esophagus	Low:1.15	Low:1.67	[54]
	Colon and rectum	Low:1.38	Low:1.27	[55]
Adherence to Mediterranean Diet (low adherence compared to medium and high adherence) Table 1	Stomach	Low adherence:1.49	Low adherence:1.49	[56]
	Colon and rectum	Low adherence:1.12	Low adherence:1.13	[57]
	Bladder	Low adherence: 1.26	Low adherence: 1.05	[58]
Alcohol consumption (High consumption compared to moderate and low consumption) Table 1	Oropharynx	High: 5.30	High: 5.70	[59]
	Esophagus	High: 4.69	High: 8.32	[59]
	Liver	High: 1.59	High: 3.89	[59]
	Colon and rectum	High:1.53	High:1.24	[59]
	Breast		High:1.61	[59]
Infection (compared to non-infected patients)	Stomach	Infected with H. Pylori: 2.89	Infected with H. Pylori: 2.89	[60]
Air pollution (exposure to PM_10_ and PM_2.5_) compared to non-exposed)	Lung	PM_10_: 1.08 PM_2.5_: 1.09	PM_10_: 1.08 PM_2.5_: 1.09	[41]

### Statistical analysis

To calculate the proportion of cases in which exposure may have played a causal role, we relied on the Population Attributable Fraction (PAF) formula that calculates the proportion by which a cancer would be reduced if a risk factor is eliminated. A gender-specific attributable fraction was calculated using the total prevalence in the total population (aged 25–64 years). The Levin formula, the classic and most common version of the PAF formula, was applied for the study [21].

The formula below is adopted in risk factors with dichotomous variables such as physical inactivity (inactive vs. moderate or highly active population), adherence to Mediterranean diet (low vs. moderate or high adherence), alcohol consumption (high vs. moderate or low), H.Pylori infection (infected population) and in air pollution (exposed population):

$$ \mathrm{PAF}=\mathrm{P}\left(\mathrm{RR}\hbox{-} 1\right)/\left(1+\mathrm{P}\left(\mathrm{RR}\hbox{-} 1\right)\right) $$

- P = proportion of population at current exposure level
- RR = the relative risk at exposure level

The above method expresses the “excess” cases- those that are the result of the risk factor- as a percentage of the total number of the corresponding cancer cases.

Alternatively, in the case where the risk factor presents more than one exposure level, such as in tobacco smoking (ex-smokers, current smokers), and BMI (overweight, obesity), (Table 1), a modified formula of the Levin formula was adopted as follows [22]:

$$ {\mathrm{P}\mathrm{AF}=\mathrm{P}}_1\left({\mathrm{RR}}_1\hbox{-} 1\right){+\mathrm{P}}_2\left({\mathrm{RR}}_2\hbox{-} 1\right)/1+\left({\mathrm{P}}_1\left({\mathrm{RR}}_1\hbox{-} 1\right)+{\mathrm{P}}_2\left({\mathrm{RR}}_2\hbox{-} 1\right)\right) $$

P_1_ and P_2_ denote the population proportion at risk of the first level (overweight) and P_2_ the population proportion at risk of the second level (obese), with RR_1_ and RR_2_ as their respective relative risks. [22]. Excess cases from both levels are aggregated to result in an overall percentage of attributable fractions for each risk factor. The number of cases resulting from these risk factors was further calculated using an estimate of the Lebanese nationals in 2018 [23]. Projected cancer incidence rates for 2018 were multiplied by the corresponding male or female population and further multiplied by the PAF for each cancer site as shown in Tables 4 and 5. Each risk factor was studied individually, knowing that meta-analyses generally use fully adjusted models for potential confounders. Inter-correlation between risk factors or unobserved risk factors was not introduced in this study analyses.

**Table 4:** Percentage attributable risk factor, relative risk, and actual incidence rates, in Males

Risk factor	Percentage attributable risk factor PAF (%) in Males in 2008	Projected incident cases in Lebanon 2018 (rate per 100,000). Data from Shamseddine et al.	Number of cases in 2018 resulting from risk factors^a^
Tobacco smoking, former smokers and current smokers	Esophagus: 43%	0.6	6
	Larynx: 74%	7.9	129
	Lung: 79%	32.7	568
	Bladder: 53%	41.2	480
	Gastric: 24%	9.3	49
	Liver: 23%	7.9	40
	Pancreas: 25%	5.9	32
Body Mass Index (BMI), overweight and obese	Colon: 22%	17.1	83
	Rectal: 19%	4.4	18
	Liver: 24%	7.9	42
	Renal cell: 20%	N/A	
	EGCA: 38%	N/A	
	Pancreatic:15%	5.9	19
Physical activity, Low activity	Gastroesophageal: 7%	N/A	
	Colorectal: 17%	Colon 17.1, Rectal	80
		4.4	
	Bladder: 4%	41.2	8
Low adherence to Mediterranean diet	Gastric: 7%	9.3	14
	Colorectal: 2%	Colon 17.1, Rectal 4.4	9
	Bladder: 4%	41.2	36
Alcohol, heavy drinkers	Oropharyngeal: 34%	3.7	28
	Esophagus: 31%	0.6	4
	Liver: 7%	7.9	12
	Colorectal:6%	Colon 17.1, Rectal 4.4	28
H. Pylori infection	Gastric: 50%	9.3	102
Air pollution^a^ (PM_2.5_ concentration above allowable levels)	Lung: 13%	32.7	94

*N/A* Data not available

^a^assuming 4.5 million Lebanese nationals in 2018

**Table 5:** Percentage attributable risk factor, relative risk, and actual incidence rates, in Females

Risk factor	Percentage attributable risk factor PAF (%) in Females in 2008	Projected incident cases in Lebanon 2018 (rate per 100,000). Data from Shamseddine et al.	Number of cases in 2018 resulting from risk factors^a^
Tobacco smoking, former smokers and current smokers	Esophagus: 33%	0.7	5
	Larynx: 65%	2.0	30
	Lung: 72%	14.3	237
	Bladder: 40%	13.4	123
	Gastric: 17%	7.7	30
	Liver: 16%	6.6	24
	Pancreas: 18%	2.3	10
	Cervix: 21%	5.8	28
Body Mass Index (BMI), overweight and obese	Colon: 6%	10.9	15
	Rectum: 3%	5.9	4
	Ovary: 9%	22.0	46
	Liver: 15%	6.6	23
	Renal cell: 29%	N/A	
	EGCA: 39%	N/A	
	Pancreas: 15%	2.3	8
	Endometrium: 32%	N/A	
	Breast: 5%	137.0	158
Physical activity, Low activity	Breast:6%	137.0	189
	Gastroesophageal:21%	N/A	
	Bladder: 7%	13.4	22
	Colorectal: 10%	Colon 10.9, Rectal 5.9	39
Low adherence to Mediterranean diet	Gastric: 6%	7.7	11
	Colorectal: 2%	Colon 10.9, Rectal	8
	Bladder: 1%	5.9	3
		13.4	
Alcohol, heavy drinkers	Oropharyngeal: 4%	2.8	3
	Esophagus: 6%	0.7	1
	Liver: 3%	6.6	5
	Colorectal:1 = 0.2%	Colon 10.9, Rectal	1
	Breast:0.5%	5.9 137.0	16
H. Pylori infection	Gastric: 50%	7.7	89
Air pollution (PM_2.5_ concentration above allowable levels)	Lung: 13%	14.3	43

*N/A* Data not available

^a^assuming 4.5 million Lebanese nationals in 2018

## Results

The analyses shown in Table 4 for males and Table 5 for females estimate the percentage and number of cancer cases that could be reduced for each risk factor 10-years following the exposure time in 2008.

Smoking recorded the highest attributable percentage among all risk factors. Lung and laryngeal cancers in both males and females had the largest proportions attributable, with 79% and 72% of lung cancer cases in males and females respectively are the result of smoking. As for the rest of the cancers associated with smoking, the percentages and relative risks were higher in males than in females (Table 4 and Table 5).

Similarly, body mass index (BMI) had a stronger effect on cancer incidence in males than in females. The effect of BMI on pancreatic and esophageal and gastric cardia (EGCA) cancer was high comparatively in both genders with 15%and 38–39%, for pancreatic and EGCA cancers respectively. GI cancers were more prevalent in obese and overweight males compared to females, as shown in liver, colon and rectal cancers (Table 4 and Table 5). High BMI was associated with a third of endometrial cancer and 9% in ovarian and 5% in breast cancer.

High physical activity in our study had a less than 20% protective effect on any of the gastroesophageal, colorectal and bladder cancers in males. Low activity was related to 21% of gastroesophageal cancer in female cases, and a smaller proportion of 6% of total breast cancer cases.

Nearly 60–70% of the Lebanese population moderately adheres to a healthy Mediterranean diet by scoring between 3 and 6 points out of 9 with no significant differences noted across genders or age groups. The minimal adherence to a healthy Mediterranean diet is expected to increase gastric cancer incidence by 6–7% in both males and females. It was related to a lesser extent to colorectal and bladder cancer in both genders.

Significant differences were noted in the drinking habits of males compared to females. In general, females were non-drinkers or occasional drinkers, while the males were moderate to heavy drinkers (Table 2). Nearly a third of the cases of oropharyngeal and esophageal cancer will be the result of heavy alcohol consumption in males, while it will account for 4% and 6% respectively in females. Heavy drinking will result in 7% and 3% of liver cancer in males and females respectively, as liver cancer is primarily linked to heavy drinking.

H.Pylori infection was prevalent in half the general Lebanese population in 2010; this is expected to result in 50% of the Gastric cancer cases in 2018.

Urban pollution is expected to contribute to 13% of the total lung cancer cases in 2018. According to the studies done in greater Beirut and specific spots at the West Beirut area [19, 20], the PM_2.5_ and PM_10_ greatly exceeded the WHO standards. The reported concentrations for PM_10_ and PM_2.5_ were 63.3 μg/ m^3^ and 20.4 μg/ m^3^ respectively, exceeding the 20 μg/ m^3^ and 10 μg/ m^3^ levels set by international standards (Table 1) [19, 20]. According to the National Health Statistics report in 2012, it is estimated that nearly 85% of the Lebanese population reside on the coastal and urban areas [24].

As shown in Table 6, the cancer with largest influences by risk factors were lung cancer with 92% and 85% of the cases induced by smoking and air pollution for males and females respectively. Upper digestive tract cancers including oropharyngeal, esophageal, and laryngeal with at least 34% of the cases in males being the result of smoking or alcohol. Influence of risk factors on GI tract cancers was more pronounced in males in colorectal cancer with more than three times higher and cancer nearly twice as high in liver cancer than their female counterparts. Gastric and EGCA cancers were comparable between both genders. Cancer incidence in females was most influenced by smoking as noted in lung cancer (85%), gastric (73%), bladder (48%) and pancreatic (33%) cancers. Female specific cancers such as breast and ovarian cancers were less influenced by these factors, with the exception of endometrial cancer, for which 32% were attributable to high BMI.

**Table 6:** Risk factors contribution to specific cancers

Cancer site	PAF (%) by risk factor Males Females		Joint hazard of risk factors
Oropharyngeal	Alcohol (34%)	Alcohol (4%)	
Esophageal	Smoking (43%) Alcohol (31%)	Smoking (33%) Alcohol (6%)	M(76%), F (37%)
Laryngeal	Smoking (74%)	Smoking (65%)	
Gastroesophageal	Low activity (7%)	Low activity (21%)	
EGCA	BMI ≥25 (38%)	BMI ≥25 (39%)	
Gastric	Smoking (24%) Low Med diet (7%), H.Pylori (50%)	Smoking (17%) Low Med diet (6%), H.Pylori (50%)	M (81%), F (73%)
Lung	Smoking (79%), Air pollution (13%)	Smoking (72%), air pollution (13%)	M (92%), F(85%)
Colorectal	BMI ≥25 (41%), Low activity (17%), Low Med diet (2%), Alcohol (6%)	BMI ≥25 (9%), Low activity (10%), Low Med diet (2%), Alcohol (0.2%)	M (66%), F(21%)
Liver	BMI ≥25 (24%), Alcohol (7%), Smoking (23%)	BMI ≥25 (15%), Alcohol (3%), Smoking (16%)	M (54%), F(34%)
Bladder	Smoking (53%), Low activity (4%), Low Med Diet (4%)	Smoking (40%), %), Low activity (7%), Low Med Diet (1%)	M (61%), F(48%)
Pancreas	BMI ≥25 (15%), Smoking (25%)	BMI ≥25 (15%), Smoking (18%)	M(40%), F(33%)
Renal Cell	BMI ≥25 (20%)	BMI ≥25 (29%)	
Breast		Low activity (6%) BMI ≥ 25 (5%), Alcohol (0.5%)	F (12%)
Ovarian		BMI ≥25 (9%)	
Endometrial		BMI ≥25 (32%)	
Cervix		Smoking (21%)	F(21%)

Based on the estimate of numbers increased due to smoking, the largest increases are expected in laryngeal, lung, and bladder cancers for males, and lung and bladder cancers for females. An expected total of 805 additional cases in 2018 will be the result of smoking. Over 180 cases of colorectal cancer will be due to high BMI and low physical activity in males. In females, high BMI and low physical activity will result in a total of 158 and 189 cases of breast cancer respectively (Tables 4 and 5).

## Discussion

Smoking remains the main culprit in lung cancer incidence due to its high chemical constituent and its impact on health in general. Our results revealed that smoking will add around 1800 cases of cancer including respiratory and GI cancers. Lebanon has one of the highest proportions of smoking in the region in males and the highest in females [25]. A direct link has been established between cigarette smoking, mortality, and lung cancer [26], controlling for genetic and demographic variability. According to a recent epidemiological study in Lebanon by Shamseddine et al. in 2014, the largest increases in cancer incidence by 2018 in Lebanon in males are expected in prostate, bladder, lung, Non-Hodgkin’s and colon cancers. As for females, breast, ovary, non-Hodgkin’s, lung and colon cancers are expected to mark the highest cancer incidence increases [7]. This projection, in line with our results, showed that smoking-related cancers including bladder and lung mark among the highest expected increases in males and lung for females, further highlighting the legacy of smoking. In addition, our analysis showed that smoking had high influence on rare cancers such as esophageal and laryngeal cancer, with incidence rate of less than 1 and 8 per 100,000 in the Lebanese general population. In addition to cigarette smoking, the WHO report [14] showed that 22% of the adult population in Lebanon are regular Arguileh (waterpipe) smokers with the majority of them aged less than 35 years, which adds to the burden of respiratory tract cancers in both sexes [14].

Our estimates revealed that over 400 diagnosed cases of GI and breast cancers in 2018 will be caused by overweight and obesity. The proportion of obese and overweight has reached an all-time high. An increase in processed and highly caloric food coupled with a sedentary lifestyle has affected a generation of young adults with increasingly higher BMI [27]. A 5 kg/m^2^ increase in BMI was associated with an increase in uterine, gallbladder, kidney and liver cancer mainly [28]. In Lebanon, 27% of the adult population is obese mostly in those aged over 35 years. High BMI will affect GI cancers, mainly in males, accounting for 41% of the male colorectal cancer cases and 24% of liver cancers. In females, our results revealed obesity was associated with a third of endometrial cancer and less with ovarian and breast cancer. Studies showed that other factors, such as reproductive history and menopausal status in the case of breast cancer are likely to influence these female cancers [29, 30].

A large number of studies have confirmed the role of physical activity in both sexes through a dose–response association, mainly in colon and breast cancer [31]. No strong evidence was shown between the protective effects of physical activity on other cancers [32]. Domestic chores explain the proportion of higher activity in females over 35 than their male counterparts. Females aged over 25 in Lebanon recorded an average of 121 min of physical activity, including a combination of walking –moderate or vigorous intensity activities per day, compared to 93 min for men; however, active men tend to engage in more vigorous types of activities [14]. Our results showed that physical activity influenced gastroesophageal cancer in both sexes yet being a rare cancer the risk reduction is unlikely to make significant changes. The increase in number of breast cancer cases resulting from high BMI and low physical activity is largely explained by the high incidence rate of breast cancer compared to other cancers.

Less than 7% of cancers studied in our population will be related to low adherence to Mediterranean diet in 2018; this can be explained by the continuing adoption of a healthy diet in the general Lebanese population. A large prospective cohort study including over 28 thousand participants in Greece between 1994 and 1999 showed that a two-point increase in Mediterranean diet adherence scale score was associated with a 12% decrease in cancer incidence while adjusting for other lifestyle habits [33]. A large review of over 100 case–control studies and a recent report from the American Institute for Cancer Research showed that adherence to Mediterranean diet protected against neoplasms notably in oral cavity, esophagus, stomach, large bowel, larynx, lung and urinary bladder [34].

Potential protection of Mediterranean diet to cancer is increased in low alcohol drinkers as compared to the heavy drinkers [35]. Alcohol acts as an antagonist to folate, necessary for DNA synthesis, thus increasing the risk of cancer in a low-folate diet [36]. Consistent evidence from a review of case control and cohort studies showed a dose–response relationship between alcohol and esophageal, colorectal, breast and liver cancers [34]. Drinking habits in the Lebanese society remain substantially lower than western societies due to social and religious conformities. Our data showed that 34% of the cases of oropharyngeal cancer will be attributable to heavy drinking in males and 4% in females in 2018. The results reflect the big differences between males and females in drinking habits and quantities consumed.

In 2002, cancers caused by infections were estimated at 17.8% of the total number of cancers worldwide [37]. The International Agency of Research on Cancer (IARC) identified H. Pylori as a Group I carcinogen and the most common causative agent in infection-related cancers [13]. H. Pylori can manifest as an asymptomatic chronic inflammation, causing stomach cancer and gastric lymphoma in 1–3% of the infections [38]. It was identified as the strongest risk factor for gastric cancer resulting in 75% of the total number of cases [39]. A representative sample of the Lebanese population revealed that 52% of adults were infected with H. Pylori [18]. Half of gastric cancer cases in Lebanon in 2018 are expected to be the result of this infection; higher proportions of H. Pylori are expected in those who are elderly or of low socioeconomic status [18]. Our calculations showed that over 190 gastric cancer cases will be the result of H. Pylori infections.

The IARC have classified PM and outdoor air pollution as a group I carcinogen related to lung cancer [40]. Fine PM of less than 10 μm- PM_10_ are more hazardous as they can penetrate and adhere to lung tissues more naturally. They are estimated to cause about 7% of total mortality of trachea, bronchus and lung cancers worldwide [40]. Growing urban population, an inefficient transport system and an inadequate national electrical power system lead to serious pollution concerns in the Middle East [41].

Currently, no trials are ongoing to assess the influence of a multitude of risk factors on cancers in our region to understand the high cancer incidence reported in Lebanon compared to its neighboring countries. Exposure to risk factors is complex and has indirect implications to different diseases; thus eliminating certain exposure now does not imply a drop in cancer cases by 2018. Past exposures keep residual effect over years such as smoking, diet, H. Pylori infection, and air pollution. Our PAF estimates lack confidence intervals as they are difficult to obtain and are beyond the scope of this paper. Results of this report showed that the highest benefits can be achieved by controlling tobacco smoking; as shown, at least two-thirds of the proportion of respiratory cancers can be reduced if smoking is eliminated.

Campaigns targeting the young population will impact smoking in general; it was revealed that the average age at smoking onset in the Lebanese population is 18 years [14]. Limited alcohol consumption would help decrease the upper GI tract cancers as shown earlier. Due to the relatively low prevalence of alcohol consumption, alcohol control in Lebanon is still indirect; thus imposing high taxes on such products and issuing stricter laws concerning drinking and driving would help limit uncontrolled drinking. The risk factors included in this study are interrelated; thus while controlling for one risk factor, an individual can be protected against other related risk factors. As an example, adherence to Mediterranean diet results in lower BMI and less alcohol, that is by just controlling diet. Due to the recent adoption of a westernized lifestyle, the new generation is more exposed to processed food; therefore awareness campaigns in schools and by general practitioners or pediatricians could help restrain this unhealthy lifestyle for both parents and children. Parents are working longer hours; in fact Lebanon has the highest female proportion in the job market compared to the Arab world, with around 31% being employed [14]. This suggests that children will increasingly depend on school lunches. A control on eating habits can be done through public health authorities imposing healthy meals for children in their schools. Another agenda item for school would be to target sedentary lifestyle among kids, by increasing outdoor activities as part of the educational curriculums. Pollution in Beirut has been increasing due to the centralization and agglomeration around the capital; cleaner gasoline and organized public transport can help reduce the smoke. The ministry of public health is ensuring access to clean water and encouraging basic sanitation habits which will further decrease H. Pylori infections.

## Conclusions

For efficient reduction of cancer incidence, our results show that smoking and obesity are the main risk factors to target; this will have the largest influence in controlling incidence across many cancers. The expected H. Pylori infection reduction will eventually result in a decrease in infection- related cancers. On the other hand, targeting diet, physical activity and pollution would have a lesser and indirect impact. However, the challenges around implementing strict laws in addition to economic and political issues in Lebanon are expected to result in slow progress over the next decade.

## Abbreviations

BMI: Body Mass Index
EGCA: Esophageal and gastric cardia
GI: Gastro intestinal
PAF: Population Attributable Fraction
PM_10_ or PM_2.5_: Particulate matter
RR: Relative Risk
WHO: World Health Organization
